# Nitrous Oxide (“Whippets”)–Induced Subacute Combined Degeneration: A Case Report

**DOI:** 10.51894/001c.164578

**Published:** 2026-07-31

**Authors:** Austin Benedict, Alexa Atty, Colin Buday, Michelle Andary, Rani Gebara

**Affiliations:** 1 University of Michigan Health - Sparrow

## 66

### Introduction

Recreational nitrous oxide (“whippets”) use is an increasingly recognized cause of functional vitamin B12 deficiency, which can lead to subacute combined degeneration (SACD) of the spinal cord. SACD is characterized by progressive neurologic dysfunction due to dorsal column involvement and peripheral neuropathy. Early recognition of nitrous oxide–related neurologic injury is important, as prompt treatment may prevent irreversible deficits.

### Case Description

A 20-year-old male with a history of recreational nitrous oxide use presented with 1–2 months of progressively worsening symmetric numbness and tingling in the distal hands and feet, which progressed proximally and was associated with gait instability. Over the preceding weeks, he developed worsening bilateral lower extremity weakness and sensory impairment requiring assistance with ambulation. Neurologic examination demonstrated preserved strength but distal sensory deficits, including loss of vibration and proprioception in a stocking-glove distribution and diminished pinprick sensation in the lower extremities. Electromyography revealed a length-dependent sensorimotor axonal and demyelinating polyneuropathy. Laboratory studies demonstrated vitamin B12 deficiency with elevated methylmalonic acid and homocysteine levels. MRI of the cervical and upper thoracic spine revealed longitudinal T2 hyperintensity within the dorsal columns consistent with SACD. The patient reported chronic nitrous oxide use for 1–2 years with increased use in the month prior to symptom onset. He was admitted, treated with aggressive vitamin B12 supplementation, counseled on cessation of nitrous oxide use, and referred for physical therapy.

### Discussion

Nitrous oxide inactivates vitamin B12 by oxidizing its cobalt core, resulting in functional B12 deficiency despite normal or mildly reduced serum levels in some cases. This disruption impairs myelin formation and leads to neurologic manifestations including peripheral neuropathy and SACD. Clinical findings often include distal paresthesias, gait instability, proprioceptive loss, and dorsal column abnormalities on MRI. Increased recreational use has led to a growing number of reported neurologic complications, highlighting the importance of screening for nitrous oxide exposure in patients with compatible neurologic presentations.

### Conclusion

Nitrous oxide misuse should be considered in patients presenting with neurologic findings consistent with subacute combined degeneration. Early recognition, cessation of exposure, and prompt vitamin B12 supplementation are essential to prevent permanent neurologic injury.

